# Supplementary material for: Feasibility and effect of high-intensity training on the progression of motor symptoms in adult individuals with Parkinson’s disease: A systematic review and meta-analysis
Source: PLoS One. 2023 Nov 10;18(11):e0293357. doi: 10.1371/journal.pone.0293357 (PMC10637666; doi:10.1371/journal.pone.0293357)
Supplement: S1 Appendix — (DOCX) [file pone.0293357.s002.docx]

# High-sensitivity search strategy

**Medline (PubMed)/Cochrane/SportDiscus**

#1 "Parkinson Disease"[Mesh] OR (Parkinson Disease) OR (Idiopathic Parkinson's Disease) OR (Lewy Body Parkinson's Disease) OR (Parkinson's Disease, Idiopathic) OR (Parkinson's Disease, Lewy Body) OR (Parkinson Disease, Idiopathic) OR (Parkinson's Disease) OR (Idiopathic Parkinson Disease) OR (Lewy Body Parkinson Disease) OR (Primary Parkinsonism) OR (Parkinsonism, Primary) OR (Paralysis Agitans)

#2 "High-Intensity Interval Training"[Mesh] OR (High-Intensity Interval Training) OR ( High Intensity Interval Training) OR (High-Intensity Interval Trainings) OR (Interval Training, High-Intensity) OR (Interval Trainings, High-Intensity) OR (Training, High- Intensity Interval) OR (Trainings, High-Intensity Interval) OR (High-Intensity Intermittent Exercise) OR (Exercise, High-Intensity Intermittent) OR (Exercises, High-Intensity Intermittent) OR (High-Intensity Intermittent Exercises) OR (Sprint Interval Training) OR (Sprint Interval Trainings) OR (High-Intensity Exercise Intervention)

#3 (randomized controlled trial[pt] OR controlled clinical trial[pt] OR randomized controlled trials[mh] OR random allocation[mh] OR double-blind method[mh] OR single- blind method[mh] OR clinical trial[pt] OR clinical trials[mh] OR ("clinical trial"[tw]) OR ((singl*[tw] OR doubl*[tw] OR trebl*[tw] OR tripl*[tw]) AND (mask*[tw] OR blind*[tw])) OR ("latin square"[tw]) OR placebos[mh] OR placebo*[tw] OR random*[tw] OR research design[mh:noexp] OR follow-up studies[mh] OR prospective studies[mh] OR cross-over studies[mh] OR control*[tw] OR prospectiv*[tw] OR volunteer*[tw]) NOT (animal[mh] NOT human[mh])

# EMBASE (Elsevier)

#1 'Parkinson disease'/exp OR (Parkinson disease) OR (idiopathic parkinsonism) OR (Lewy bodies of Parkinson disease) OR (Lewy bodies of Parkinson's disease) OR (Lewy bodies of Parkinsons disease) OR (Lewy body Parkinson disease) OR (Lewy body Parkinson's disease) OR (Lewy body Parkinsons disease) OR (paralysis agitans) OR (Parkinson dementia complex) OR (Parkinsons disease) OR (Parkinsons disease) OR (primary parkinsonism)

#2 'high intensity interval training'/exp (high intensity interval training) OR (high-intensity intermittent exercise) OR (high-intensity intermittent training) OR (high-intensity interval exercise) OR (high-intensity interval training) OR (HIIE exercise) OR (HIIT) OR (intermittent high-intensity training) OR (interval high-intensity training) OR (High-Intensity Exercise Intervention)

#3 'crossover procedure'/exp AND [embase]/lim OR ('prospective study'/exp AND [embase]/lim) OR ('follow up'/exp AND [embase]/lim) OR ('placebo'/exp AND [embase]/lim) OR ('clinical trial'/exp AND [embase]/lim) OR ('single blind procedure'/exp AND [embase]/lim) OR ('double blind procedure'/exp AND [embase]/lim) OR

('randomization'/exp AND [embase]/lim) OR ('controlled clinical trial'/exp AND [embase]/lim) OR ('randomized controlled trial'/exp AND [embase]/lim)

# BVS

#1 MH: "Parkinson Disease" OR (Doença de Parkinson) OR (Parkinson Disease) OR (Enfermedad de Parkinson) OR (Doença de Parkinson Idiopática) OR (Doença de Parkinson com Corpos de Lewy) OR (Mal de Parkinson) OR (Paralisia Agitante) OR (Parkinsonismo Primário) OR MH: C10.228.140.079.862.500 OR MH: C10.228.662.600.400 OR MH:

C10.574.928.750

#2 MH: "High-Intensity Interval Training" OR (Treinamento Intervalado de Alta Intensidade) OR (High-Intensity Interval Training) OR (Entrenamiento de Intervalos de Alta Intensidad) OR (Exercício Intermitente de Alta Intensidade) OR (Treinamento Intervalado de Arranque) OR (High-Intensity Exercise Intervention) OR MH: G11.427.410.698.277.311.25 OR MH: I03.350.311.250
