# Supplementary material for: Feasibility and effect of high-intensity training on the progression of motor symptoms in adult individuals with Parkinson’s disease: A systematic review and meta-analysis
Source: PLoS One. 2023 Nov 10;18(11):e0293357. doi: 10.1371/journal.pone.0293357 (PMC10637666; doi:10.1371/journal.pone.0293357)
Supplement: S2 Appendix — (DOCX) [file pone.0293357.s003.docx]

**Appendix B.** GRADE certainty evidence of outcomes

| **Outcomes** | **Anticipated absolute effects^*^ (95% CI)** | **№ of participants (studies)** | **Certainty of the evidence (GRADE)** |
| --- | --- | --- | --- |
|  | **Risk with [high intensity]** |  |  |
| UPDRS | MD **4.74 lower** (6.1 lower to 3.39 lower) | 335 (7 RCTs) | ⨁⨁⨁⨁ High |
| TUG | SMD **0.79 SD lower** (1.13 lower to 0.46 lower) | 148 (2 RCTs) | ⨁⨁⨁◯ Moderate |
| Maximum oxygen consumption | MD **2.17 higher** (1.51 higher to 2.82 higher) | 277 (5 RCTs) | ⨁◯◯◯ Very low |
| Quality of life | MD **1.29 lower** (2.08 lower to 0.5 lower) | 459 (5 RCTs) | ⨁⨁⨁◯ Moderate |

Note: ***The risk in the intervention group** (and its 95% confidence interval) is based on the assumed risk in the comparison group and the **relative effect** of the intervention (and its 95% CI). **CI:** confidence interval; **MD:** mean difference; **SMD:** standardised mean difference

**GRADE Working Group grades of evidence**

**High certainty:** we are very confident that the true effect lies close to that of the estimate of the effect.

**Moderate certainty:** we are moderately confident in the effect estimate: the true effect is likely to be close to the estimate of the effect, but there is a possibility that it is substantially different.

**Low certainty:** our confidence in the effect estimate is limited: the true effect may be substantially different from the estimate of the effect.

**Very low certainty:** we have very little confidence in the effect estimate: the true effect is likely to be substantially different from the estimate of effect.
