# Supplementary material for: Feasibility and effect of high-intensity training on the progression of motor symptoms in adult individuals with Parkinson’s disease: A systematic review and meta-analysis
Source: PLoS One. 2023 Nov 10;18(11):e0293357. doi: 10.1371/journal.pone.0293357 (PMC10637666; doi:10.1371/journal.pone.0293357)
Supplement: Attachment — Submitted filename: Response Letter_revised manuscript.pdf [file pone.0293357.s005.pdf]

Response Letter,

Dear editor, we thank you for the excellent comments of the reviewers and for the opportunity we have been given to revise the manuscript, making its quality better and within the standards required by PLOS ONE. Below are the answers to each question:

Questions

Addressing a substantial amount of grammatical mistakes found throughout the manuscript. An example is provided here from page 10

Answer: Thank you, we sent this manuscript to English review.

Reviewers also mention that the authors should carefully delineate differences in delaying disease progression vs improving motor function. Refer to reviewer comments for details.

Answer: Thank you, we change it.

The authors also must give more attention and comparison to outcomes obtained from moderate exercise practice in patients. At present, most of the comparisons made to the high-intensity studies are "control groups". There are numerous studies of PD patients engaging in moderate intensity exercise with positive motor outcomes and some of these show positive motor outcomes from moderate exercise. Here are but a few of such studies.

Shulman LM, et al. 2013. Randomized clinical trial of 3 types of physical exercise for patients with Parkinson disease. JAMA Neurol 1169 70, 183-190.

Ridgel AL, et al. 2009. Forced, not voluntary, exercise improves motor function in Parkinson's disease patients. Neurorehabiol Neural Repair 23, 600-608.

Kanegusuku H, et al. 2021. Influence of motor impairment on exercise capacity and quality of life in patients with Parkinson disease. J Exerc Rehabil 17, 241-246.

Salvatore MF, et al., 2022. Establishing equivalent aerobic exercise parameters between early-stage Parkinson's disease and Pink1 knockout rats. J Parkinsons Dis 12: 1897-1915.

Answer: We appreciate this comment and we have added this paragraph and these recommended references: However, moderate-intensity exercise may be a viable option for facilitating adherence and improving exercise enjoyment for Parkinson's disease[71–73]. In literature low- or moderate-intensity exercise produced the greatest improvements in gait speed and cardiovascular fitness in humans and model animals diagnosed with Parkinson's disease [41,74,75].

The authors should have a brief discussion on whether high-intensity exercise may pose a significant barrier for exercise participation, particularly with attention the disease stage, thus leaving moderate intensity as a viable option.

Answer: Thank you for all suggestions. We added this information in discussion: In addition, previous studies have found that high-intensity training (HIT) produces health improvements, although the intensity required may be intimidating, which may not be sufficient to facilitate intrinsic motivation for exercise adherence [71]. Exercise intensity is an important variable to dose and individualize the exercise stimulus for specific participants. However, moderate-intensity exercise may be a viable option for facilitating adherence and improving exercise enjoyment for Parkinson’s disease [71–73].

Reviewer #1

1. This is a very important topic and a comprehensive review of all high-intensity articles would be an important contribution to the literature. I did not do more than skim this article however because it is not written in clearly understandable English. I would be happy to review a revision after the authors have worked with someone to fully translate into English. There were some terms like "lower members" where I can make guesses about what the authors mean - but really have no idea and thus think it's likely best to revise for readability first prior to reviewers devoting more time on the details. I also noted when downloading the PDF that this manuscript has been under review before and prior reviewers were also concerned about English language readability--among other concerns with which I agreed (definition of high-intensity exercise)

Answer: We regret this and ask for your understanding. This manuscript has been peer-reviewed. In addition, we reviewed all studies and excluded six studies because they didn't report details about HIIT.

2. Should the authors choose to revise and resubmit I would recommend including in the discussion some practical next step suggestions for real-world implementation of these findings. They acknowledge in the introduction that it's a confusing space because methodology so different -- but what common threads can we draw to produce clear real-world exercise guidelines? To some degree the Parkinson's Foundation has already done this (see their publicly available table for PROVIDERS re exercise prescription on their website; and there is an accompanying journal article either just published or under review).

Answer: Thank you. We added this information in discussion: In addition, previous studies have found that high-intensity training (HIT) produces health improvements, although the intensity required may be intimidating, which may not be sufficient to facilitate intrinsic motivation for exercise adherence [71]. Exercise intensity is an important variable to dose and individualize the exercise stimulus for specific participants. However, moderate-intensity exercise may be a viable option for facilitating adherence and improving exercise enjoyment for Parkinson's disease [71–73].

Reviewer #2

1. This resubmitted manuscript by De sena et al is a review of the role of intense exercise in patients with PD. The authors responded to the initial critique, so it's unclear as to why an additional reviewer was needed for the manuscript. Regardless, this reviewer has a few additional comments.

Answer: Thank you, we tried to improve this paper based on suggestions.

2. The grammar still needs to be improved.

Answer: Thank you, we sent this manuscript to English review.

3. Abstract: in the very last line, the authors indicate that high intensity exercise can delay the progression of the disease. The authors present no compelling evidence for such an effect on the disease progression itself. This is an over statement of the findings. Intense exercise appears to improve motor function (perhaps even delaying motor dysfunction), but this is far different from delaying the progression of the disease. The holy grail in PD treatment is recovery in nigrostriatal dopamine levels/function. The authors present no data suggesting that there is any dopamine recovery with exercise and as far as this reviewer is aware, no such data are available in the human literature. In preclinical animal studies, our lab has clearly shown motor improvement that is totally independent of nigrostriatal dopamine recovery (Churchill et al, 2017, Sconce et al., 2015, Hood et al, 2016) using a recovery/restoration study design. Therefore, the authors need to eliminate the phrase, 'delay disease progression', not only in the abstract but also on pages 2 (4th paragraph), page 12 (first full paragraph), and page 13 (last paragraph).

Answer: Thank you, we agree, and it was removed in all paper.

4.Page 9: lower 'members' functionality. The use of the word, members, is a bit odd. I assume the authors mean 'limb'? This should also be corrected at the bottom of page 11.

Answer: We agree, and it was change; the information was wrong.
