# Supplementary material for: Feasibility and effect of high-intensity training on the progression of motor symptoms in adult individuals with Parkinson’s disease: A systematic review and meta-analysis
Source: PLoS One. 2023 Nov 10;18(11):e0293357. doi: 10.1371/journal.pone.0293357 (PMC10637666; doi:10.1371/journal.pone.0293357)
Supplement: Attachment — Submitted filename: Reviewer#1e2_comments.docx [file pone.0293357.s004.docx]

| Reviewers #1 | ANSWERS |
| --- | --- |
| Overview and general recommendation:  Reviewer #1: Title: Feasibility of high-intensity training and its effects on the progression of motor symptoms in  adult individuals with Parkinson's disease: a systematic review and meta-analysis.  General comments: The authors performed an interesting systematic review and meta-analysis about the  feasibility and the effects of high-intensity exercise training on the motor symptoms of people with Parkinson's  disease. Although the methodology used to perform the systematic review and collect the articles is correct, I  believe there are critical flaws with the manuscript in its current form that need to be addressed before publication.  The authors are also recommended to revise the text for English writing entirely.  The authors performed a good job replying all questions raised by the reviewers. But I have a couple of minor  points. Firstly, please consider including a rationale in the discussion benchmarking the current papers on  prescription of exercise with your findings (PMID: 35194464) and including some message also in discussion on  the Covid pandemic including some data on PD and Pandemic (PMID: 35043063; PMID: 32304118) | Thank you very much for reviewing our manuscript, and for providing helpful suggestions that strengthen our work. Below we address all the comments and questions, and provide a summary after changes to the manuscript. In addition, please note that we reviewed all grammar issue and made a few changes to the systematic review to be consistent with what is recommended in the Cochrane Handbook. We also re-ran the search strategy to make sure that our results are still current. All suggestions were in red lines. |
| Major comment: |  |
| The most critical methodological flaw, in my view, was the author's decision to define high-intensity exercise. The  authors stated, "Based on the guidelines of the American College of Sports Medicine (ACSM), aerobic exercises  for the elderly, when performed above 64% VO2peak, are considered high-intensity exercises (ACMS, 2014)". The  current classification, however, is not in line with the ACSM guidelines, where high-intensity exercise is defined as  reported below:  > 60%HRR or VO2R  > 64% VO2max  > 76 HRmax  * VO2peak is not used!  Using this correct classification (ACSM, 2014), the majority of the selected studies should not be included in the  current study. This unfortunate aspect directly affects the study findings and their originality since most prior  reports have systematically examined the effects of moderate-intensity exercise interventions on motor symptoms  in people with Parkinson's disease. | We agree with this appointment. However, we followed other references that observed and excluded eight studies with different intensity. In addition, we made some changes and following this consensus:  According to studies exercise intensity is often measured using methods such as heart rate (HR), heart rate reserve (HRR) or oxygen uptake (VO2). There are several types of exercise and intensity, additionally, high-intensity is defined and categorized as vigorous effort (70%–90% of peak HR; 60%–84% of HRR; 60%–79% of peak VO2) or “very hard” effort (≥ 90% of peak HR; ≥ 85% of HRR; ≥ 80% of peak VO2)[17–19].   1. Marriott CFS, Petrella AFM, Marriott ECS, Boa Sorte Silva NC, Petrella RJ. High-Intensity Interval Training in Older Adults: a Scoping Review. Sports Med Open. 2021 Jul 19;7(1):49. doi: 10.1186/s40798-021-00344-4. PMID: 34279765; PMCID: PMC8289951. 2. L Vanhees, N Geladas, D Hansen, E Kouidi, J Niebauer, Ž Reiner, V Cornelissen, S Adamopoulos, E Prescott, M Börjesson, (on behalf of the writing group), Importance of characteristics and modalities of physical activity and exercise in the management of cardiovascular health in individuals with cardiovascular risk factors: recommendations from the EACPR (Part II), European Journal of Preventive Cardiology, Volume 19, Issue 5, 1 October 2012, Pages 1005–1033, |
| The second critical point is the readability of the manuscript. The text is full of grammar errors and typos, with the  structure of the paragraphs being confusing and hard to read. The authors, in my view, are recommended to  revise the English and improve the readability of the document. Some minor examples are:  - And when it is a progressive neurodegenerative disorder, any interference that modify the disease course and  benefit these individuals, such as physical exercise, need to be well informed.  - High intensity training has shown the more benefits that the practice of moderate intensity for improvements in  health in populations as obese and hypertensive  Table 1. 8 semanas/3 vezes | Thanks for suggestions. We made all. |
| We note that the grant information you provided in the ‘Funding Information’ and ‘Financial Disclosure’ sections  do not match. | Thanks for suggestions. We changed this. |
| Thank you for stating the following financial disclosure:  Funding agency: Only the corresponding author received financial support from the Higher Education Personnel  Improvement Coordination - Brazil (CAPES) - Financing Code 001, for this research.  Please state what role the funders took in the study. If the funders had no role, please state: "The funders had no  role in study design, data collection and analysis, decision to publish, or preparation of the manuscript."  If this statement is not correct you must amend it as needed.  Please include this amended Role of Funder statement in your cover letter; we will change the online submission  form on your behalf. | Thanks for suggestions. We changed this. |
| Thank you for stating the following in the Acknowledgments Section of your manuscript:  Only the corresponding author received financial support from the Higher Education Personnel Improvement  Coordination - Brazil (CAPES) - Financing Code 001, for this research.  Please note that funding information should not appear in the Acknowledgments section or other areas of your  manuscript. We will only publish funding information present in the Funding Statement section of the online  submission form.  Please remove any funding-related text from the manuscript and let us know how you would like to update your  Funding Statement. Currently, your Funding Statement reads as follows:  Funding agency: Only the corresponding author received financial support from the Higher Education Personnel  Improvement Coordination - Brazil (CAPES) - Financing Code 001, for this research.  Please include your amended statements within your cover letter; we will change the online submission form on  your behalf. | Thanks for suggestions. We changed this. |
| Reviewer #2 |  |
| Reviewer #2: Title: Feasibility of high-intensity training and its effects on the progression of motor symptoms  in adult individuals with Parkinson's disease: a systematic review and meta-analysis  This article seems well built and brings evidence of a physiological phenomenon not yet fully understood and that  certainly, deserves further study.  Some points of revision, suggested to the authors to increase the level of the paper, are provided below | We agree and added this information |
| Abstract  In Background: The objective of this review…..please to add “systematic review and meta-analysis”  In Methods and analysis: please to add “systematic review and meta-analysis”  …Members : please check this term which members!! | We changed this |
| In the main document the references should be updated according to the journal guideline  ….VO2 more than VO2 | We changed this |
| About the high-intensity physical training the authors need to analyze/shown/compare the difference training  intensity….. | Thanks for suggestions. We made all. |
| In Strategy and search databases used….. “SportDiscus and SCOPUS” | Thanks for suggestions. We made all. |
| The figure should be improved with an higher resolution <600 DPI | Thanks for suggestions. We made all. |
