# Supplementary material for: Feasibility and effect of high-intensity training on the progression of motor symptoms in adult individuals with Parkinson’s disease: A systematic review and meta-analysis
Source: PLoS One. 2023 Nov 10;18(11):e0293357. doi: 10.1371/journal.pone.0293357 (PMC10637666; doi:10.1371/journal.pone.0293357)
Supplement: Attachment — Submitted filename: Response to Reviewers.docx [file pone.0293357.s006.docx]

| Reviewers #1 | ANSWERS |
| --- | --- |
| Reviewer #1: The manuscript is improved from prior however still has some English language mistakes, incomplete sentences and overall would benefit tremendously from tighter writing. I get the gist of what they are trying to accomplish from the introduction.  The clarification of high vs moderate exercise and HIIT Is helpful. The tables are great and very useful. Likely the tables are the biggest contribution to the literature-it is helpful to have the studies summarized in such a way. | Thank you very much for reviewing our manuscript, and for providing helpful suggestions that strengthen our work. Below we address all the comments and questions and provide a summary after changes to the manuscript. In addition, please note that we reviewed all grammar issue and made a few changes. All suggestions were in red lines. |
| Major comment: |  |
| But the conclusions drawn in the discussion are a bit far reaching/too broad. I defer to the editors of PLOS One if this paper is up to standard for publishing in your journal. The content is important. But in my opinion the writing is not tight enough for publication in which you want to make it as easy as possible for your reader to seamlessly grasp the point. | We agree with this appointment. In addition, we made some changes. |
| Just a few specific examples as I was reading the paper:  This is not a full sentence: Based on symptoms, pharmacotherapy is the primary intervention for treating the disease, but its adverse effects and reduced efficacy over time suggest implementing therapies which modify the course of the disease and protect neurons against damage resulting from  neurodegeneration [8–10].  This sentence doesn’t make sense to me: However, the effectiveness of these exercises when faced with delays in motor symptoms progressing to more advanced stages of the disease has come to be questioned [9,15,16].  This is not a complete sentence: Studies suggest that if interventions (such as the practice of physical exercise) which may modify the course of the disease and benefit individuals with progressive neurodegenerative disease, they need to be well informed [28].  Our meta-analysis also showed a significant improvement in the quality of life of individuals submitted to high intensity exercise when compared to those who did not perform any exercise,  submitted is the wrong word here.  IN discussion would use word FEASIBLE rather than viable. Last sentence of first paragraph of discussion-I think they mean “adjuvant treatment to PHARMACOLOGICAL (not non-pharam) treatment”  Last sentence of conclusion in first person is completely unnecessary. | Thanks for suggestions. We made all. |
| Reviewer #2 |  |
| No comments |  |
